# Prevalence of allergic fungal sinusitis among patients with nasal polyps

**DOI:** 10.5144/0256-4947.2009.212

**Published:** 2009

**Authors:** Laila M. Telmesani

**Affiliations:** From the Department of Ear, Nose and Throat, King Faisal University, Al-Khobar, Saudi Arabia

## Abstract

**BACKGROUND:**

Nasal polyposis is a common problem in the eastern province of Saudi Arabia. Since allergic fungal sinusitis (AFS) can present with unilateral or bilateral nasal polyps, it is important to be aware of the prevalence of AFS in patients with nasal polyps.

**PATIENTS AND METHODS:**

The medical records of 91 patients with nasal polyps admitted for functional endoscopic sinus surgery were reviewed. The diagnosis of AFS was considered if histopathology showed the presence of eosinophillic mucin-containing hyphae. The following data were collected: presence of associated asthma, IgE levels, grading of CT scan findings, and operative details.

**RESULTS:**

Histopathological diagnosis was positive for AFS in 11 of 91 patients 12.1%. There was a highly significant statistical difference in IgE levels between patients suffering AFS and chronic hyperplastic rhinosinusitis with nasal polyps (*P*<.0001). The overall incidence of recurrence of polypi was 50.5% (45 of 89 patients who had follow up for a minimum of one year) and the rate of recurrence in patients with AFS was 54.5% (6 of 11 patients with AFS). There was a significant direct relationship between the CT grading of nasal polyps and recurrence, with a recurrence rate of 60.7% (34 of 56) in patients with grade III nasal polyps.

**CONCLUSION:**

This study showed that the prevalence of AFS among patients with nasal polyps is 12.1%, and suggests that CT grading of nasal polyps can be used as a prognostic factor for disease recurrence.

Allergic fungal sinusitis (AFS) is a type I hypersensitivity reaction to fungal antigens in which patients usually present with unilateral or bilateral nasal polyps.^1^ It is important to be aware of the prevalence of AFS in patients with nasal polyps. Ferguson stated that the simplest and most straight-forward requirement for defining patients as having AFS is the presence of eosinophilic mucin with hyphae.^2^ Kuhn and Javer studied whether specific IgE to fungal species was superior to total IgE in predicting persistence or recurrence.^3^ They found that total IgE was generally most sensitive. Asthma associated with AFS is estimated to range from 20% to 40%.^4^ Bee-See et al found a prevalence of AFS in adult Malaysians with chronic rhinosinusitis of 26.7% and a prevalence of associated asthma of 25%.^5^ In another study performed by Chen et al, AFS was several-fold higher in the southern compared with the northern United States.^6^ As the prevalence of AFS among patients with nasal polyps has not been estimated in the Middle East and due to the potential for disease complication in cases of AFS, we decided it was important to find the prevalence of this disease among patients with nasal polyps.

## PATIENTS AND METHODS

Between January 2004 and May 2007, 95 patients were admitted to King Fahd Hospital of the University with a diagnosis of unilateral or bilateral nasal polyps for functional endoscopic sinus surgery (FESS). The following data were collected; age, sex, CT scan findings, IgE level, operative details, final diagnosis based on histopathological findings of the removed tissue specimens and mucin (when present), recurrence in patients that were followed up for one year or more, presence of associated asthma, and the results of fungal cultures in suspected cases of AFS.

CT scan staging of nasal polyps was based on the staging of Lildholdt et al^7^ in which nasal polyps are classified according to a fixed anatomical landmark, namely the middle and inferior turbinates (grade 1: small polyps extend just below the free edge of the middle turbinate; grade 2: medium size polyps reaching between the upper and lower edges of the inferior turbinate and grade 3: large polyps reaching below the lower edge of the inferior turbinate. AFS was considered if the histopathology confirmed the presence of eosinophilic mucin-containing hyphae.^8^

Data was analyzed using SPSS version 15. For descriptive statistics, the mean and standard deviation are shown. For comparisons, the t test for independent variables, analysis of variance and chi-square were used as appropriate. The level of significance was set at <.05.

## RESULTS

The medical records of 91 patients were included in this retrospective study as the remaining 4 records were missing most of the data. The 91 patients ranged in age from 11 to 62 years with a mean (SD) age of 37.1 (13.2) years. There were 30 females (33.0%) and 61 males (67.0%). CT scan grading was available for all patients. Based on the final histopathology report, the incidence of AFS among patients with nasal polyps was 12.1% (11/91 patients). The remaining 80 patients showed histopathological features of chronic hyperplastic rhinosinusitis. An estimated IgE level was found in 77 records. The mean (SD) IgE level was 242.1 (381.3) IU/mL for patients with chronic rhinosinusitis (66 patients), and 820.2 (752.8) IU/mL for AFS patients (11 patients). An independent sample test showed a highly significant difference (*P*<.0001).

Eighty-nine patients were followed up for one year or more with mean of 16 months. The overall recurrence rate in these patients was 50.5% (45 patients). The recurrence rate in patients with AFS was 54.5% (6 of 11 patients), while the rate of recurrence in non-fungal nasal polyposis was 50% (39 of 78 patients) (Table 1).

**Table 1:** CT scan grading and recurrence of polyps after 1 year or more of follow-up in 91 patients with nasal polyposis.

	CT scan grading, n (%)			Total
Patients	I	II	III	
All^a^	1 (1.098)	32 (35.2)	58 (63.7)	91
Recurrence^b^	0	11 of 32 (34.4)	34 of 56 (60.7)^c^	45*
Non-fungal polyposis^a^	1 (1.282)	26 (33.3)	51 (65.4)	78
Recurrence^b^	0	8 of 26 (30.8)	31 of 51 (60.8)	39
Allergic fungal sinusitis^a^	0	6 (54.5)	5 (45.5)	11
Recurrence^b^	0	3 of 6 (50%)	3 of 5 (60%)	6

a Percentage of totals in right column.

b Percentage of total for grade category.

c 34/56;2 patients lost to follow-up.

* *P*<.05 across row

Sixty-six patients (72.5%) were non-asthmatic and 25 patients (27.5%) were asthmatic. Only one patient with AFS had an associated asthma (9% of all patients with AFS). Statistically, it was found that asthma is not a significant factor in nasal polyposis or AFS (*P*>.05). In 8 of 11 cases of AFS *Aspergillus* species were demonstrated in fungal cultures. In the remaining 3 cases, cultures were negative.

## DISCUSSION

Nasal polyps are a common problem with different etiologies. It is important to try to estimate the incidence of AFS among patients with nasal polyps. In this retrospective study we were able to collect data from the medical records of 91 patients admitted with the preoperative diagnosis of nasal polyps in the period from January 2004 to May 2007 for endoscopic sinus surgery. A histopathologically valid diagnosis was found in the 91 patients medical records and 11 patients (12.1%) were proven to have AFS. Bee-See et al (2005) found that the incidence of AFS in adult Malaysian patients with refractory rhinosinusitis was 26.7%, the presence of allergic mucin was the tool for diagnosing AFS, although they found fungus in secretions in only 5 patients (16.7%) and in nasal secretions and in surgical specimens in 11 (36.7%).^5^ On the other hand, Braun et al (2003) found an incidence of AFS among patients with nasal polyposis of 7%.^9^ This variability in incidence may be due to different techniques in diagnosis and differences in geographical distribution.

Our study showed that the total IgE was significantly higher in patients with AFS than those with chronic rhinosinusitis. These results are in agreement with Ferguson, who stated that total IgE in patients with AFS is frequently elevated with a mean of 668 IU/mL. with the normal being less than 125 IU/mL.^10^ Moreover, Schbert found that total IgE correlated significantly with the severity of the disease, and that an increase of 10% or more in total serum IgE during the follow-up period was a strong predictor of recurrence and the need for surgical intervention.^11^

Although we think that total IgE is important in the diagnosis and prognosis of AFS, we recognize that the large standard deviation makes it difficult to use it as a diagnostic tool. This marked variability may in part be attributed to fluctuation in disease activity. However, total IgE might be helpful to differentiate between eosinophilic mucin rhinosinusitis and AFS, where there are elevated levels in cases of AFS.^1^

In this study, the overall recurrence rate for nasal polyps was 50.5% (45 out of 89 patients). Six of 11 patients (54.5%) with AFS showed recurrence within one year, while the recurrence rate in patients with hyperplastic rhinosinusitis was 50% (39 of 78 patients). This was found to be insignificant statistically. In a retrospective study, Wynn and Har found a recurrence rate in patients with nasal polyps of 60% after 40 months of follow up.^12^ The lower rate in our study may be due to the shorter period of follow-up. Although Ferguson reported that the recurrence of AFS following surgery alone is very high,^2^ we found no accurate figure for the rate of recurrence for AFS in the literature. In applying the grading system for nasal polyps described by Lildholdt et al^7^ we found that there was a significant increase in the rate of recurrence with the increase in grade, suggesting that grading system could be used for prediction of recurrence of nasal polyps.

Asthma was found to be associated with nasal polyps in 25 patients (27.5%) and only in one patient with AFS (9%), suggesting that asthma is not an important associated pathology in allergic fungal sinusitis. In most of the literature, the rate of associated asthma in cases of AFS ranges from 30% to 40%. The lower incidence of associated asthma in our study may be due to the study having few patients to provide an accurate incidence of associated asthma. If asthma is not an important associated pathology, the high incidence of associated asthma in the literature may be related to the mixing of AFS and non-fungal eosinophillic mucin rhinosinusitis in which the incidence of associated asthma is 93%.^10^

Aspergillus was the only species found in 8 of the 11 cases diagnosed as AFS. These results are in agreement with the results of Ferguson et al, who noted that there appears to be a geographical variability in the incidence of AFS and fungal species associated with the disease process. In the United States, dematiaceous fungi are the most common while Aspergillus species cause most cases reported in the Middle East.^13^ In conclusion, this study showed that the prevalence of AFS among patients with nasal polyps is 12.1%, and IgE level was higher in patients with AFS than those with chronic hyperplastic rhinosinusitis. It suggests that CT grading of nasal polyps can be used as a prognostic factor for disease recurrence and asthma is not significantly associated with nasal polyps.
